# Supplementary material for: Prevalence of urogenital and intestinal schistosomiasis among school children in South-west Nigeria
Source: PLoS Negl Trop Dis. 2021 Jul 27;15(7):e0009628. doi: 10.1371/journal.pntd.0009628 (PMC8345861; doi:10.1371/journal.pntd.0009628)
Supplement: Attachment — Submitted filename: Reviewer - PNTD-D-20-01655_reviewer.pdf [file pntd.0009628.s001.pdf]

PLOS Neglected Tropical Diseases

Epidemiology of mixed urogenital and intestinal schistosomiasis among school children in two endemic communities of Southern Nigeria

--Manuscript Draft--

**Manuscript Number:**	PNTD-D-20-01655
**Full Title:**	Epidemiology of mixed urogenital and intestinal schistosomiasis among school children in two endemic communities of Southern Nigeria
**Short Title:**	Mixed urogenital and intestinal schistosomiasis among school children in Nigeria
**Article Type:**	Research Article
**Keywords:**	schistosomiasis, Schistosoma haematobium, Schistosoma mansoni, ectopic eggs, mixed infection, Nigeria
**Abstract:**	Background: The risk of co-infection with Schistosoma haematobium and S. mansoni and the potential harmful effect on morbidity and control is enhanced by the overlapping distribution of both species in sub-Saharan Africa. Despite the reported high endemicity of S. haematobium and S. mansoni in Nigeria, studies on the spread and impact of their mixed infection are limited. We present the analysis of mixed S. haematobium and S. mansoni infection and ectopic egg elimination of schistosome egg in school children in Osun State Nigeria.<br>Methods : The presence of the S. haematobium egg was detected in urine using the urine filtration technique while S. mansoni was detected in stool using Kato–Katz thick smear.<br>Results: A total of 466 (211 (45.3%) males vs. 255 (54.7%) females; mean age 11.6 ± 3.16 years) primary and secondary school children were enrolled for the study. The overall prevalence of schistosomiasis was 40% (185/466) with 19% (89/466) recording single S. haematobium infection (geometric egg count = 189.4 egg/10ml urine; 95% CI: range 115.9-262.9), and 9% (41/465) recording single S. mansoni infection (geometric egg count = 115.7 epg; 95% CI: range 78.4-152.9). The prevalence of ectopic S mansoni was 4.7%, while no ectopic S. haematobium was recorded. Mixed infection of S. haematobium / S. mansoni had a prevalence of 9.5% (44/466). More females (54.5%) presented with S. haematobium / S. mansoni co-infection. For both parasites, males had higher infection intensity, with significant difference observed with S. haematobium (p=0.0004). Hematuria was significant in individuals with single S. haematobium infection (p=0.002), mixed ectopic S. haematobium / S. mansoni (p=0.009) and mixed S. haematobium / S. mansoni /ectopic S. mansoni (p=0.0003).<br>Conclusions: These findings suggest the probability of interspecific interactions between S. haematobium and S. mansoni. Scaling up of mass administration of praziquantel and control measures in the study areas is highly desirable.
**Additional Information:**	
**Question**	**Response**
**Financial Disclosure**<br><br>Enter a financial disclosure statement that describes the sources of funding for the work included in this submission. Review the submission guidelines for detailed requirements. View published research articles from *PLOS NTDs* for specific examples.<br><br>This statement is required for submission and **will appear in the published article** if	The author(s) received no specific funding for this work

the submission is accepted. Please make sure it is accurate.<br><br>**Unfunded studies**<br><br>Enter: *The author(s) received no specific funding for this work.*<br><br>**Funded studies**<br><br>Enter a statement with the following details:<br>• Initials of the authors who received each award<br>• Grant numbers awarded to each author<br>• The full name of each funder<br>• URL of each funder website<br>• Did the sponsors or funders play any role in the study design, data collection and analysis, decision to publish, or preparation of the manuscript?<br>• **NO** - Include this sentence at the end of your statement: *The funders had no role in study design, data collection and analysis, decision to publish, or preparation of the manuscript.*<br>• **YES** - Specify the role(s) played.<br><br>* typeset	
**Competing Interests**<br><br>Use the instructions below to enter a competing interest statement for this submission. On behalf of all authors, disclose any competing interests that could be perceived to bias this work—acknowledging all financial support and any other relevant financial or non-financial competing interests.<br><br>This statement **will appear in the published article** if the submission is accepted. Please make sure it is accurate. View published research articles	The authors have declared that no competing interests exist.

from *PLOS NTDs* for specific examples.<br><br>**NO authors have competing interests**<br><br>Enter: *The authors have declared that no competing interests exist.*<br><br>**Authors with competing interests**<br><br>Enter competing interest details beginning with this statement:<br><br>*I have read the journal's policy and the authors of this manuscript have the following competing interests: [insert competing interests here]*<br><br>* typeset	
**Data Availability**<br><br>Authors are required to make all data underlying the findings described fully available, without restriction, and from the time of publication. PLOS allows rare exceptions to address legal and ethical concerns. See the PLOS Data Policy and FAQ for detailed information.<br><br>A Data Availability Statement describing where the data can be found is required at submission. Your answers to this question constitute the Data Availability Statement and **will be published in the article**, if accepted.	Yes - all data are fully available without restriction

**Important:** Stating 'data available on request from the author' is not sufficient. If your data are only available upon request, select 'No' for the first question and explain your exceptional situation in the text box.<br><br>Do the authors confirm that all data underlying the findings described in their manuscript are fully available without restriction?	
**Describe where the data may be found in full sentences. If you are copying our sample text, replace any instances of XXX with the appropriate details.**<br><br>• If the data are **held or will be held in a public repository**, include URLs, accession numbers or DOIs. If this information will only be available after acceptance, indicate this by ticking the box below. For example: *All XXX files are available from the XXX database (accession number(s) XXX, XXX.).*<br>• If the data are all contained **within the manuscript and/or Supporting Information files**, enter the following: *All relevant data are within the manuscript and its Supporting Information files.*<br>• If neither of these applies but you are able to provide **details of access elsewhere**, with or without limitations, please do so. For example:<br><br>*Data cannot be shared publicly because of [XXX]. Data are available from the XXX Institutional Data Access / Ethics Committee (contact via XXX) for researchers who meet the criteria for access to confidential data.*<br><br>*The data underlying the results presented in the study are available*	The data that support the findings of this study are available from the corresponding author, [OO], upon reasonable request

from (include the name of the third party and contact information or URL).<br>• This text is appropriate if the data are owned by a third party and authors do not have permission to share the data.<br><br>* typeset	
Additional data availability information:	

Manuscript

Click here to access/download;Manuscript;Ojo et al_ectopic shisto_V1.docx

Epidemiology of mixed urogenital and intestinal schistosomiasis among school children in two endemic communities of Southern Nigeria

Johnson A. Ojo[1], Samuel A. Adedokun[1], Akeem A. Akindele[1], Adedolapo B. Olorunfemi[1], Olawumi A. Otutu[1], Taiwo A. Ojurongbe[2], Bolaji N. Thomas[3], Thirumalaisamy P. Velavan[4,5]‡, Olusola Ojurongbe[1]‡.

[1]Department of Medical Microbiology & Parasitology, Ladoke Akintola University of Technology, Osogbo, Nigeria

[2]Department of Mathematical Sciences, Osun State University, Osogbo, Nigeria

[3]Department of Biomedical Sciences, College of Health Sciences and Technology, Rochester Institute of Technology, Rochester NY, USA

[4]Institute of Tropical Medicine, University of Tübingen, Germany

[5]Duy Tan University, Da Nang, Vietnam

‡Joint senior author

Corresponding author

Olusola Ojurongbe

Email: oojurongbe@lautech.edu.ng

ABSTRACT

Background: The risk of co-infection with *Schistosoma haematobium* and *S. mansoni* and the potential harmful effect on morbidity and control is enhanced by the overlapping distribution of both species in sub-Saharan Africa. Despite the reported high endemicity of *S. haematobium* and *S. mansoni* in Nigeria, studies on the spread and impact of their mixed infection are limited. We present the analysis of mixed *S. haematobium* and *S. mansoni* infection and ectopic egg elimination of schistosome egg in school children in Osun State Nigeria.

Methods: The presence of the *S. haematobium* egg was detected in urine using the urine filtration technique while *S. mansoni* was detected in stool using Kato–Katz thick smear.

Results: A total of 466 (211 (45.3%) males vs. 255 (54.7%) females; mean age 11.6 ± 3.16 years) primary and secondary school children were enrolled for the study. The overall prevalence of schistosomiasis was 40% (185/466) with 19% (89/466) recording single *S. haematobium* infection (geometric egg count = 189.4 egg/10ml urine; 95% CI: range 115.9-262.9), and 9% (41/465) recording single *S. mansoni* infection (geometric egg count = 115.7 epg; 95% CI: range 78.4-152.9). The prevalence of ectopic *S mansoni* was 4.7%, while no ectopic *S. haematobium* was recorded. Mixed infection of *S. haematobium*/*S. mansoni* had a prevalence of 9.5% (44/466). More females (54.5%) presented with *S. haematobium*/*S. mansoni* co-infection. For both parasites, males had higher infection intensity, with significant difference observed with *S. haematobium* (p=0.0004). Hematuria was significant in individuals with single *S. haematobium* infection (p=0.002), mixed ectopic *S. haematobium*/*S. mansoni* (p=0.009) and mixed *S. haematobium*/*S. mansoni*/ectopic *S. mansoni* (p=0.0003).

Conclusions: These findings suggest the probability of interspecific interactions between *S. haematobium* and *S. mansoni.* Scaling up of mass administration of praziquantel and control measures in the study areas is highly desirable.

Keywords: schistosomiasis, *Schistosoma haematobium*, *Schistosoma mansoni*, ectopic eggs, mixed infection, Nigeria

INTRODUCTION

Schistosomiasis, a neglected tropical diseases targeted for elimination by the World Health Organization [1], is a major public health problem, with Nigeria [2–5], ranking first among African countries with the highest disease burden [6]. The disease is caused by parasites of the genus *Schistosoma* and is responsible for the most obvious reduction in age-standardized years lived with disability (YLD) between 2006 and 2016 [7]. The most affected group are the school-aged children, involved with one water contact activity or the other that brings them in contact with the free-swimming cercariae, released from infected snail species in freshwater [4] [8]. The disease is present in 78 countries, affecting more than 250 million people annually, and presenting with two major forms; a urogenital disease caused by *S. haematobium* and an intestinal disease caused by *S. mansoni, S. japonicum, S. mekongi,* and *S. intercalatum*. *S. haematobium* (*Sh*) and *S. mansoni* (*Sm*) are the two major species endemic in sub-Saharan Africa, along with a few cases of *S. intercalatum*, localized in rain forest areas of Central Africa [1].[9]

Urogenital schistosomiasis is associated with pathological outcomes, such as hematuria, bladder cancer, and hydronephrosis, while chronic intestinal disease is characterized by hepatomegaly, splenomegaly, and progressive periportal fibrosis resulting in portal hypertension, esophageal varices, liver surface irregularities, portal-systemic venous shunts, and hematemesis [9,10]. The impaired physical and cognitive development arising from chronic infection among children is a major concern in many parts of the world [13]. The risk of co-infection with *Sh* and *Sm* is greatly enhanced by the overlapping distribution of both species in Africa [11]. However, there is a paucity of information on the determinants, distribution and the impact of such mixed infections on endemic populations. Results from co-infection in experimental models show the two species could form heterologous male–female pairs, with the male carrying the female to its preferred site for oviposition and the female producing eggs characteristic of her species in an uncharacteristic site [15,16]. This phenomenon referred to as

hybridization is believed to be responsible for ectopic egg elimination resulting in the detection of *Sm* eggs in urine or *Sh* eggs in feces, in areas where mixed infection occur [14, 15], and suggestive of possible sexual interactions in nature between *Sh* and *Sm*. Nevertheless, hybridization which was not previously reported from human schistosomes species in Africa, was recently observed in France from a patient originally from Côte d'Ivoire [16, 17]. It is believed that disease epidemiology and phenotypic characteristics could be altered by hybridization, which ultimately could affect transmission and host compatibility of the parasite [18, 19].

An increasing number of foci where co-infections between *Sh* and *Sm* occurs has been reported in some parts of Africa [14, 20–22]. In some of these foci, differences in schistosomiasis-associated morbidity as well as infection intensity has been reported between single and mixed infections [22]. Understanding the epidemiology of mixed infections therefore, will help us to answer important standing questions on the underlying mechanisms towards morbidity and the development of effective strategies for the prevention and control of schistosomiasis in co-endemic areas. Both *Sh* and *Sm* occur in Nigeria with *Sh* having higher prevalence. While quite a number of studies have reported the occurrence of both species in the same foci there has been no information in the possible ectopic egg elimination and its impact on infection intensity. In this report, we present results from a cross-sectional study conducted to investigate possible mixed *Schistosoma* infections and associated disease covariates in two schistosomiasis endemic communities in Nigeria.

Material and methods

Study site

This study was conducted among school children (age 4–19 years), recruited from Ore and Ilie communities, Osun State, Nigeria. Urine and stool samples were collected from the primary and

secondary school pupils in these communities, who consented or whose parents/guardian gave consent to participate in this study. The two communities are located very closely on latitude 4°34’ and 4°36’E, and Longitude 7°56’ and 7°58’N, and only separated by a dam in the rain forest zone. The dam owned and managed by the State Water Corporation, Olorunda local government area, southwest Nigeria is believed to be the breeding site of *Schistosoma* in the area owing to abundant presence of snail intermediate host in the dam. These communities depend on the dam for their domestic water supply, fishing and other water related activities. These communities were chosen because of the previous reports of schistosome endemicity [23], and a the presence of the dam.

Sample Collection

The sample size was obtained, using the formula for a cross-sectional study [24]. Using a prior prevalence of 37.5% among school children positive for schistosomiasis [23], a marginal error of 5% and a type 1 error of 5%, a minimum sample of 289 school children was needed. In all, 466 school children participated in this study. Individual demographic information was collected with a structured questionnaire, while two sterile, universal containers, individually labelled for urine and stool collection, were distributed to consenting school children. Instructions on proper urine and stool collection procedure was given to the students; for each participant, one urine and stool samples were collected.

Parasitological examination

The presence of the *Sh* egg was detected using the urine filtration technique, as previously described [4]. Briefly, 10 ml of the freshly passed urine sample was pushed through a micro-filter membrane of 10–12 µm (MF, Whatman, New Jersey, USA) using a syringe. The micro-filter membrane was then carefully placed on a glass slide, mounted on a microscope and examined using a low-power objective (10×) of a light microscope. For stool analysis, two Kato–

Katz thick smear were prepared using 41.7 mg template of the stool material each and microscopically examined for *Sm* and other intestinal parasites [25]. Slides were examined by two independent and experienced scientists. For quality control, 15% of all positive and negative slides were re-examined by a third independent microscopist who was blinded of the results of the first two scientist. The *Sh* infection intensity was expressed as the number of eggs detected in 10ml of urine (eggs/10ml) while the *Sm* infection intensity was expressed as the number of eggs detected per gram of feces (epg). The counted eggs were categorized into light infection (1–99 epg for *Sm* and 1–49eggs/10ml for *Sh*), moderate (100–399 epg for *Sm*) and heavy infections (≥400epg for *Sm* and ≥ 50 eggs/10ml for *Sh*) [26]. Single infection was defined as passing eggs of only one species, and mixed infection as passing eggs of both *Sm* and *Sh*. The incidence of ectopic egg excretion was measured qualitatively (positive/negative). Ectopic egg elimination refers to the elimination of schistosomal eggs via the unusual route–i.e. *Sh* eggs in feces or *Sm* eggs in urine. Overall *Sm* infection refers to both mixed and single *Sm* infections. Overall *Sh* infection includes both mixed and single *Sh* infections. Each child found to be positive for any of the schistosome species was treated with 40mg/kg praziquantel by the study team.

Ethics

The human protocol reported in this study was carried out in accordance with the 1964 Helsinki declaration and met institutional ethics of Ladoke Akintola University College of Medicine. Approval for this project was obtained from the Ethical Review Committee, Osun State Ministry of Health (approval number OSHREC/PRS/569T/131) and informed consent received from every participant or guardian before they were recruited into the study.

Data analysis

Data were double entered into an excel sheet, cleaned and then analyzed using IBM Statistical Package for Social Sciences (SPSS) for Windows version 20 (SPSS, Inc., city, country). Data were described using percentages, geometric means and 95% confidence interval. The egg output data was 10 log-transformed for the purpose of normalizing skewed egg distribution. Geometric means of egg count (GM epg or eggs/10 ml) were computed for microscopically positive individuals and intensity of infection analyzed. The χ-square test was used to evaluate association between infection status (*Sm*, *Sh* and mixed infection) and disease covariates (sex, age etc). The independent-samples T-test was used to compare GM infection intensities with age and sex.

RESULTS

Complete sample comprising of about 5gm of feces and 10 ml of urine from each participants were obtained from 466 primary and secondary school children. They consisted of 211 (45.3%) males and 255 (54.7%) females with a mean age of 11.6±3.16 years. The overall mean weight and height of the participants are 31.2±9.60kg and 1.41±0.78m, respectively. The breakdown of the infection according to age group showed that older age group (12-19 year) were generally more infected except for children that has mixed ectopic *Sh* infection. In all the age group comparison, no significant difference was observed. Similarly, no significant difference was observed between gender and infection prevalence, with females having higher proportion of *Sh* (57.3%), *Sm* (58.5%) single infections, and *Sh* /*Sm* mixed infection (54.5%). For the mixed infections of ectopic *Sm*/*Sh* and ectopic *Sm/Sh/Sm*, males had higher prevalence but with no significant difference (Table 1). 33.7% of the participants positive for *Sh* had blood in their urine compared to 66.3% that were positive, but without blood in their urine, and the difference was statistically significant (*p=0.002)*. Similarly, the proportion of the mixed infections of ectopic *Sm*/*Sh* (*p=0.009)* and ectopic *Sm*/*Sh/Sm* (*p=0.0003)* had a statistically significant effect on the proportion of participants with blood in urine. The mean weight and the mean height of the study

population is shown in Table 1. Mean weight and mean heights of positive and negative participants showed no statistically significant difference. The overall prevalence of schistosomiasis in the study was 40% (185/466). Single *Sh* infection among the participants was 19% (89/466) with a geometric egg count of 189.4egg/10mls (95%CI: 115.9-262.9) while 9% (41/465) had single *Sm* infection with a geometric egg count of 115.7 epg (95%CI: 78.4-152.9). Mixed *Sh/Sm* infection was recorded in 9.5% (44/466) of the study population. Mixed ectopic *Sm* occurring along with *Sh* (Figure 1) was recorded in 4.5% (21/466) of the study population while 1(0.2%) participant had single ectopic *Sm* infection. The occurrence and distribution of *Schistosoma* infection is shown in Table 2 with an overall prevalence of 31% and 10% for *Sh* and *Sm,* respectively.

The association between ectopic *Sm* egg elimination and infection intensities of *Sh* and *Sm* is shown in Table 3. High prevalence of ectopic *Sm* egg was observed in high infection intensities of both *Sh* (18%) and *Sm* (15.6%) producing a strong significant association in both cases. Figure 2 shows the relationship between age prevalence and infection intensity in the study population. Age group 12-19 years recorded higher prevalence of *Sh*, *Sm* and ectopic *Sm* infection compared to the younger age group (4-11 years), but the difference in all cases was not statistically significant. In both *Sh* and *Sm*, the younger age group (4-11 years) had higher infection intensity and was statistically significant (p=0.016) in the *Sm* group. For the ectopic *Sm* infection group, the pattern was different as the older age group recorded the higher infection intensity but the difference was not statistically significant (Figure 2).

The relationship between sex and infection intensity in the study population is shown in Figure 3. Females were more infected with both *Sh* and *Sm*, but the difference was not statistically significant. On the other hand, male recorded more ectopic *Sm* infection but the difference was not statistically significant. In both *Sh* and *Sm*, males had higher infection intensity and the

difference was significant in those infected with *Sh* (p=0.0004). In ectopic *Sm*, females had higher infection intensity but the difference was not statistically significantly (Figure 3).

Discussion

We present the analysis of mixed *Sh* and *Sm* infections and the ectopic egg elimination of schistosome egg in school children in Osun State Nigeria. The study revealed a high prevalence of both *Sh* and *Sm* infections among school children in Ilie and Ore communities of Osun State Nigeria. The overall prevalence of schistosomiasis was 40% and as expected the prevalence of *Sh* (31%) was significantly higher than *Sm* (10%) (p>0.05). Earlier report had shown widespread urinary schistosomiasis in the Niger River basin, the Southwest, the Central and Northern highlands, and around Lake Chad while intestinal schistosomiasis was less prevalent but also wide spread in Nigeria [27]. The various reports across the different regions of Nigeria had reported the co-occurrence of both *Sh* and *Sm* infection with prevalence ranging from 60.8 to 4.8% and 8.9 to 2.9%, respectively [28–31]. The high prevalence of both *Sh* and *Sm* reported in this study reflects the high exposure of the pupils to contaminated water body that harbor the cercariae of both *Sh* and *Sm* and the possible ongoing control challenges in this area. Also more worrisome is the observation that 10% of the school children were co-infected with both urogenital and intestinal schistosomiasis. Inter-specific parasite interactions in areas with mixed species infections have been predicted to have a significant impact on host morbidity. For example lower liver morbidity has been reported in individuals with mixed infection compared to those with single *Sm* infections and higher bladder morbidity reported in those with mixed compared to those with single *Sh* infections [22]. The lowering impact of liver morbidity in individuals with mixed infections was suggested to be caused by the hybrid eggs produced by the mating of *Sh* males with *Sm* females with the deposition of such eggs in the urinary oviposition site (ectopic egg elimination) thereby reducing the amount of classical *Sm* eggs that are capable of inducing liver morbidity [22, 32]. While we observed *Sm* eggs in urine in our

study, *Sh* egg were not recovered in stool. By implication, with the high prevalence and high intensity of *Sh* in our study area, co-infection with *Sm* may aggravate the associated *Sh* bladder morbidity. To clarify this observation future study must investigate the impact of mixed *Sh* and *Sm* co-infections on both liver and bladder morbidity as well as other schistosomes related clinical manifestations in the study area.

A relatively high prevalence (4.7%) of lateral spine egg (*S. mansoni*) ectopic excretion was observed in the urine of participants in this study. Ectopic egg elimination (*Sh* eggs in feces and *Sm* eggs in urine) has been reported in endemic areas where both schistosome co-exist [14, 33]. This phenomenon has been linked to parasite hybridization resulting from closely related sister species of schistosomes. For example *Schistosoma bovis* that causes intestinal schistosomiasis in ruminants is closely related to *Sh* and hybridization between *Sh* and *S bovis*, has been reported in Senegal [34, 35] and also linked to the outbreak of schistosomiasis in Corsica, France [18]. Similarly, hybridization between the two major human schistosomes, *Sh* and *Sm* which used to be very rare or not taken into consideration, possibly because of the assumption of the significant phylogenetic distance, has now been described in Senegal [36] and in France in a patient that originated from Côte d'Ivoire [17]. Although our study did not conduct hybridization study, the elimination of ectopic *Sm* could imply hybridization between the two human schistosomes as previously reported in Cameroon [14, 15] that may warrant further investigation. Emergence of hybrids may impact negatively on schistosomiasis control as they are well adapted to intermediate hosts, able to modify the epidemiology of the disease [16, 37, 38] and spread to new areas and become invasive populations [18].

The age-related prevalence of schistosomiasis has been shown to increase as the age increases peaking in adolescence and lowering among adults [39]. Unfortunately, adults were not included in this study making it impossible to investigate this age-infection profile.

Nevertheless, the adolescence group was significantly more infected, but had lower intensity of infection compared to the younger age group in this study. The older children are engaged in more water contact activity leading to the observed higher prevalent but possess longer history of exposure and higher parasite-specific acquired immunity leading to lower infection intensity [39]. The prevalence of *Sh* and *Sm* was higher in females, and the male students on the other hand had higher prevalence of mixed infections. Both *Sh* and *Sm* recorded higher infection intensity in male students, while for mixed infection the infection intensity was higher in female students. Previous studies have documented heavier infection in males than females in *Sh* and *Sm* contrary to what was observed in this study [40, 41] although others have agreed with this observation [42, 43]. Socio-cultural or behavioral factors focusing mainly on differences in the water contact pattern between males than females are generally implicated in the frequently observed gender-related differences in prevalence and infection intensity [4] although susceptibility factors like hormonal differences and genetic factors cannot be ruled out. The explanation for the differences in gender-related prevalence in this study may not be precise, but we may speculate that the females had higher water contact activities with a considerable longer duration of body exposure. The higher infection intensity observed in males may warrant further investigation as it generally believed that high testosterone levels in males will significantly lower the infection prevalence and intensity [44]. Since this is not the focus of our study, it is clear that more studies will be needed to actually decipher the impact of gender on infection prevalence and intensity in our study area.

A close association was observed between hematuria and the presence of *Sh* eggs in the urine, similar to the reports of Ekpo et al. (2010) [5]. The close relationship between hematuria and presence of eggs in the urine could be explored for the assessment of urinary schistosomiasis in communities. Consequently, the collection of urine specimens and their examination may not

be necessary in the classification of communities according to the level of endemicity of urinary schistosomiasis.

Understanding the exact relation between mixed infection and infection intensity is crucial, as increased egg loads can have important repercussions on the development of morbidity [11]. Higher *Sh* and *Sm* infection intensities recorded in mixed than in single infections and a positive association between *Sh* and *Sm* infections was reported in this study. While some studies have reported higher infection intensities in mixed infections [33, 45], other studies on a larger scale have reported inconsistent results [46, 47]. Possibly, the relationship between mixed infection and infection intensity varies according to local differences in *Sm* and *Sh* transmission. Larger sample size in different locations might be needed to accurately decipher the influence of mixed schistosome infection on the infection intensity.

In summary, this study reveals the presence of high prevalence of mixed *Sh* and *Sm;* and ectopic *Sm* eggs elimination in Ilie and Ore communities of Osun State Nigeria. The results of this study indicates that some form of inter-specific interactions exist between *Sh* and *Sm*, and may produce a potentially important consequences for the development of morbidity in the study areas. Further study on the impact of mixed *Sh* and *Sm* infections on both liver and bladder morbidities as well as scaling up of mass administration of praziquantel and control efforts in the study areas is highly desirable.

Acknowledgment

The authors are sincerely grateful to all consenting participants, the parents of the participants and all the teachers for their co-operation. The image of the egg of *Schistosoma* was captured with the assistance of Dr. Adrian Streit in his laboratory at Max Planck Institute for Evolutionary Biology, Tuebingen

References

[1] WHO. Schistosomiasis, https://www.who.int/news-room/fact-sheets/detail/schistosomiasis (2020, accessed 11 May 2020).

[2] Bolaji O, Adeyeba O, Ojurongbe O, et al. Epidemiological studies on Urinary Schistosomiasis in Osun State,Nigeria by Bolaji et al., www.ijprs.com. *Int J Pharm Res Sch* 2014; 3: 655–662.

[3] Ezeh CO, Onyekwelu KC, Akinwale OP, et al. Urinary schistosomiasis in Nigeria: a 50 year review of prevalence, distribution and disease burden. *Parasite Paris Fr* 2019; 26: 19.

[4] Ojurongbe O, Sina-Agbaje OR, Busari A, et al. Efficacy of praziquantel in the treatment of Schistosoma haematobium infection among school-age children in rural communities of Abeokuta, Nigeria. *Infect Dis Poverty* 2014; 3: 30.

[5] Ekpo UF, Laja-Deile A, Oluwole AS, et al. Urinary schistosomiasis among preschool children in a rural community near Abeokuta, Nigeria. *Parasit Vectors* 2010; 3: 58.

[6] Steinmann P, Keiser J, Bos R, et al. Schistosomiasis and water resources development: systematic review, meta-analysis, and estimates of people at risk. *Lancet Infect Dis* 2006; 6: 411–425.

[7] King CH, Galvani AP. Underestimation of the global burden of schistosomiasis. *The Lancet* 2018; 391: 307–308.

[8] Ciddio M, Mari L, Sokolow SH, et al. The spatial spread of schistosomiasis: A multidimensional network model applied to Saint-Louis region, Senegal. *Adv Water Resour* 2017; 108: 406–415.

[9] Kabore A, Ibikounle M, Tougoue JJ, et al. Initiating NTD programs targeting schistosomiasis and soil-transmitted helminthiasis in two provinces of the Democratic Republic of the Congo: Establishment of baseline prevalence for mass drug administration. *Acta Trop* 2017; 166: 177–185.

[10] King CH, Dangerfield-Cha M. The unacknowledged impact of chronic schistosomiasis. *Chronic Illn* 2008; 4: 65–79.

[11] Gryseels B, Polman K, Clerinx J, et al. Human schistosomiasis. *Lancet Lond Engl* 2006; 368: 1106–1118.

[12] Khalil SB, Mansour NS. Worm development in hamsters infected with unisex and cross-mated Schistosoma mansoni and Schistosoma haematobium. *J Parasitol* 1995; 81: 8–11.

[13] Webster BL, Southgate VR, Tchuem Tchuenté LA. Mating interactions between Schistosoma haematobium and S. mansoni. *J Helminthol* 1999; 73: 351–356.

[14] Cunin P, Tchuem Tchuenté L-A, Poste B, et al. Interactions between Schistosoma haematobium and Schistosoma mansoni in humans in north Cameroon. *Trop Med Int Health TM IH* 2003; 8: 1110–1117.

[15] Ratard RC, Ndamkou CN, Kouemeni LE, et al. Schistosoma mansoni eggs in urine. *J Trop Med Hyg* 1991; 94: 348–351.

[16] Depaquit J, Akhoundi M, Haouchine D, et al. No limit in interspecific hybridization in schistosomes: observation from a case report. *Parasite Paris Fr* 2019; 26: 10.

[17] Le Govic Y, Kincaid-Smith J, Allienne J-F, et al. Schistosoma haematobium-Schistosoma mansoni Hybrid Parasite in Migrant Boy, France, 2017. *Emerg Infect Dis* 2019; 25: 365–367.

[18] Boissier J, Grech-Angelini S, Webster BL, et al. Outbreak of urogenital schistosomiasis in Corsica (France): an epidemiological case study. *Lancet Infect Dis* 2016; 16: 971–979.

[19] Tchuem Tchuenté L-A, Southgate VR, Jourdane J, et al. Schistosoma intercalatum: an endangered species in Cameroon? *Trends Parasitol* 2003; 19: 389–393.

[20] Abdel-Wahab MF, Yosery A, Narooz S, et al. Is Schistosoma mansoni replacing Schistosoma haematobium in the Fayoum? *Am J Trop Med Hyg* 1993; 49: 697–700.

[21] Garba A, Labbo R, Tohon Z, et al. Emergence of Schistosoma mansoni in the Niger River valley, Niger. *Trans R Soc Trop Med Hyg* 2004; 98: 296–298.

[22] Koukounari A, Donnelly CA, Sacko M, et al. The impact of single versus mixed schistosome species infections on liver, spleen and bladder morbidity within Malian children pre- and post-praziquantel treatment. *BMC Infect Dis* 2010; 10: 1–14.

[23] Awosolu OB, Shariman YZ, Haziqah M T F, et al. Will Nigerians Win the War Against Urinary Schistosomiasis? Prevalence, Intensity, Risk Factors and Knowledge Assessment among Some Rural Communities in Southwestern Nigeria. *Pathog Basel Switz*; 9. Epub ahead of print 17 February 2020. DOI: 10.3390/pathogens9020128.

[24] Charan J, Biswas T. How to Calculate Sample Size for Different Study Designs in Medical Research? *Indian J Psychol Med* 2013; 35: 121–126.

[25] Katz N, Chaves A, Pellegrino J. A simple device for quantitative stool thick-smear technique in Schistosomiasis mansoni. *Rev Inst Med Trop Sao Paulo* 1972; 14: 397–400.

[26] Coulibaly JT, N'Gbesso YK, N'Guessan NA, et al. Epidemiology of Schistosomiasis in Two High-Risk Communities of South Côte d'Ivoire with Particular Emphasis on Pre-School–Aged Children. *Am J Trop Med Hyg* 2013; 89: 32–41.

[27] Jones I. The History of Schistosomiasis in Nigeria. *http://schisto.stanford.edu/pdf/Nigeria.pdf*, 2015, p. 2.

[28] Adeniran AA, Mogaji HO, Aladesida AA, et al. Schistosomiasis, intestinal helminthiasis and nutritional status among preschool-aged children in sub-urban communities of Abeokuta, Southwest, Nigeria. *BMC Res Notes* 2017; 10: 637.

[29] Aribodor DN, Bassey SA, Yoonuan T, et al. Analysis of Schistosomiasis and soil-transmitted helminths mixed infections among pupils in Enugu State, Nigeria: Implications for control. *Infect Dis Health* 2019; 24: 98–106.

[30] Dawaki S, Al-Mekhlafi HM, Ithoi I, et al. PREVALENCE AND RISK FACTORS OF SCHISTOSOMIASIS AMONG HAUSA COMMUNITIES IN KANO STATE, NIGERIA. *Rev Inst Med Trop Sao Paulo* 2016; 58: 54.

[31] Singh K, Muddasiru D, Singh J. Current status of schistosomiasis in Sokoto, Nigeria. *Parasite Epidemiol Control* 2016; 1: 239–244.

[32] Meurs L, Mbow M, Vereecken K, et al. Bladder Morbidity and Hepatic Fibrosis in Mixed Schistosoma haematobium and S. mansoni Infections: A Population-Wide Study in Northern Senegal. *PLoS Negl Trop Dis*; 6. Epub ahead of print 27 September 2012. DOI: 10.1371/journal.pntd.0001829.

[33] Meurs L, Mbow M, Vereecken K, et al. Epidemiology of mixed Schistosoma mansoni and Schistosoma haematobium infections in northern Senegal. *Int J Parasitol* 2012; 42: 305–311.

[34] Huyse T, Webster BL, Geldof S, et al. Bidirectional Introgressive Hybridization between a Cattle and Human Schistosome Species. *PLOS Pathog* 2009; 5: e1000571.

[35] Webster BL, Diaw OT, Seye MM, et al. Introgressive hybridization of Schistosoma haematobium group species in Senegal: species barrier break down between ruminant and human schistosomes. *PLoS Negl Trop Dis* 2013; 7: e2110.

[36] Huyse T, Van den Broeck F, Hellemans B, et al. Hybridisation between the two major African schistosome species of humans. *Int J Parasitol* 2013; 43: 687–689.

[37] Boon NAM, Van Den Broeck F, Faye D, et al. Barcoding hybrids: heterogeneous distribution of Schistosoma haematobium × Schistosoma bovis hybrids across the Senegal River Basin. *Parasitology* 2018; 145: 634–645.

[38] Sene-Wade M, Marchand B, Rollinson D, et al. Urogenital schistosomiasis and hybridization between Schistosoma haematobium and Schistosoma bovis in adults living in Richard-Toll, Senegal. *Parasitology* 2018; 145: 1723–1726.

[39] Mutapi F, Burchmore R, Mduluza T, et al. Age-related and infection intensity-related shifts in antibody recognition of defined protein antigens in a schistosome-exposed population. *J Infect Dis* 2008; 198: 167–175.

[40] Kabatereine NB, Kemijumbi J, Ouma JH, et al. Epidemiology and morbidity of Schistosoma mansoni infection in a fishing community along Lake Albert in Uganda. *Trans R Soc Trop Med Hyg* 2004; 98: 711–718.

[41] Moira AP de, Fulford AJC, Kabatereine NB, et al. Analysis of Complex Patterns of Human Exposure and Immunity to Schistosomiasis mansoni: The Influence of Age, Sex, Ethnicity and IgE. *PLoS Negl Trop Dis*; 4. Epub ahead of print September 2010. DOI: 10.1371/journal.pntd.0000820.

[42] Goselle NO, Anegbe D, Imandeh GN, et al. Schistosoma mansoni infections amongst school children in Jos, Nigeria. *Sci World J*; 5. Epub ahead of print 2010. DOI: 10.4314/swj.v5i1.61485.

[43] Tefera A, Belay T, Bajiro M. Epidemiology of Schistosoma mansoni infection and associated risk factors among school children attending primary schools nearby rivers in Jimma town, an urban setting, Southwest Ethiopia. *PloS One* 2020; 15: e0228007.

[44] Nakazawa M, Fantappie MR, Freeman GL, et al. Schistosoma mansoni: susceptibility differences between male and female mice can be mediated by testosterone during early infection. *Exp Parasitol* 1997; 85: 233–240.

[45] Robert CF, Bouvier S, Rougemont A. Epidemiology of schistosomiasis in the riverine population of Lagdo Lake, northern Cameroon: mixed infections and ethnic factors. *Trop Med Parasitol Off Organ Dtsch Tropenmedizinische Ges Dtsch Ges Tech Zusammenarbeit GTZ* 1989; 40: 153–158.

[46] Ahmed ES, Daffalla A, Christensen NO, et al. Patterns of infection and transmission of human schistosomiasis mansoni and schistosomiasis haematobium in White Nile Province, Sudan. *Ann Trop Med Parasitol* 1996; 90: 173–180.

[47] Booth M, Mayombana C, Kilima P. The population biology and epidemiology of schistosome and geohelminth infections among schoolchildren in Tanzania. *Trans R Soc Trop Med Hyg* 1998; 92: 491–495.

Table 1: General characteristics and prevalence of human schistosomiasis in the study population

Key: *Sh*: *Schistosoma haematobium*; *Sm*: *Schistosoma mansoni*; E*Sm*: Ectopic *Schistosoma mansoni*

Table 2: Schistosomal infection prevalence and intensities

Key: *Sh*: *Schistosoma haematobium*; *Sm*: *Schistosoma mansoni*; E*Sm*: Ectopic *Schistosoma mansoni*

Table 3: Relation between ectopic Schistosoma mansoni eggs in urine and intensities of S. haematobium egg in urine and S.mansoni egg in stool.

Figure 1: Ectopic egg elimination of *S. mansoni* in the urine of one of the study participants: **A**: *S. mansoni* egg**; B, C, D**: *S. haematobium*.

Figure 2: Age-prevalence and -intensity curves for schistosomias. The bars indicate overall infection prevalence per age group. Lines indicate mean log-transformed infection intensities among positive subjects. **A**: *S. haematobium* infection; Age vs prevalence $p>0.05$; infection intensity vs age $p=0.55$. **B:** *S. mansoni* infection; Age vs prevalence **p=0.016***; infection intensity vs age p=0.33. **C:** Ectopic egg *(S. mansoni* in urine) infection; Age vs prevalence $p>0.05$; infection intensity vs age $p=0.059$

Figure 3: Sex-prevalence and -intensity curves for schistosomias. The bars indicate overall infection prevalence per sex. Lines indicate mean log-transformed infection intensities among positive subjects. **A**: *S. haematobium* infection; Sex vs prevalence $p=0.487$; infection intensity vs sex ***p*=0.004***. **B:** *S. mansoni* infection; Age vs prevalence $p=0.883$; infection intensity vs age p=0.797. **C:** Ectopic egg *(S. mansoni* in urine) infection; Age vs prevalence $p=0.073$; infection intensity vs age $p=0.289$

Click here to access/download;Figure;Ojo et al_Figure 1.tif

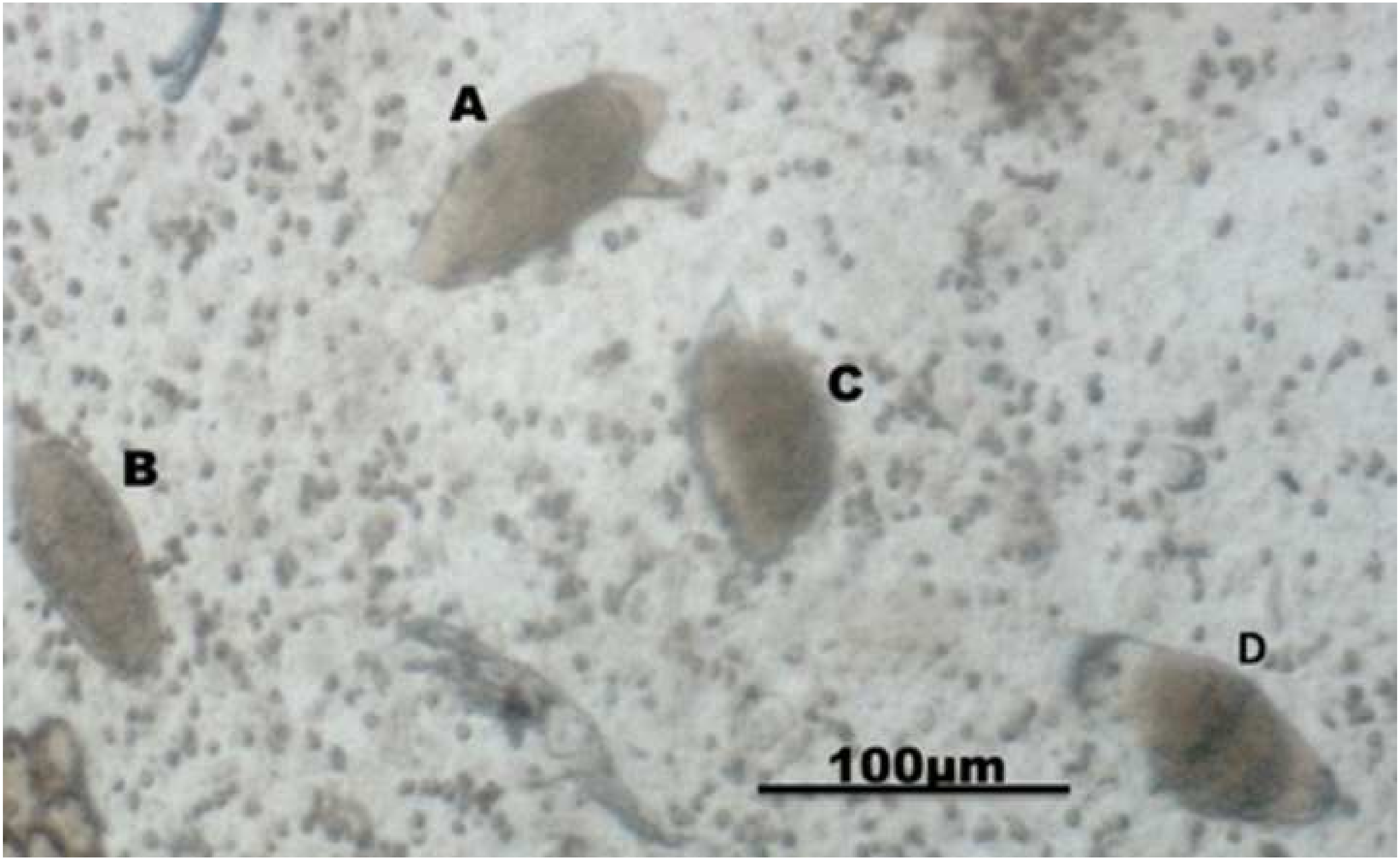

Figure 2

Click here to access/download;Figure;Ojo et al_Figure 2.tif

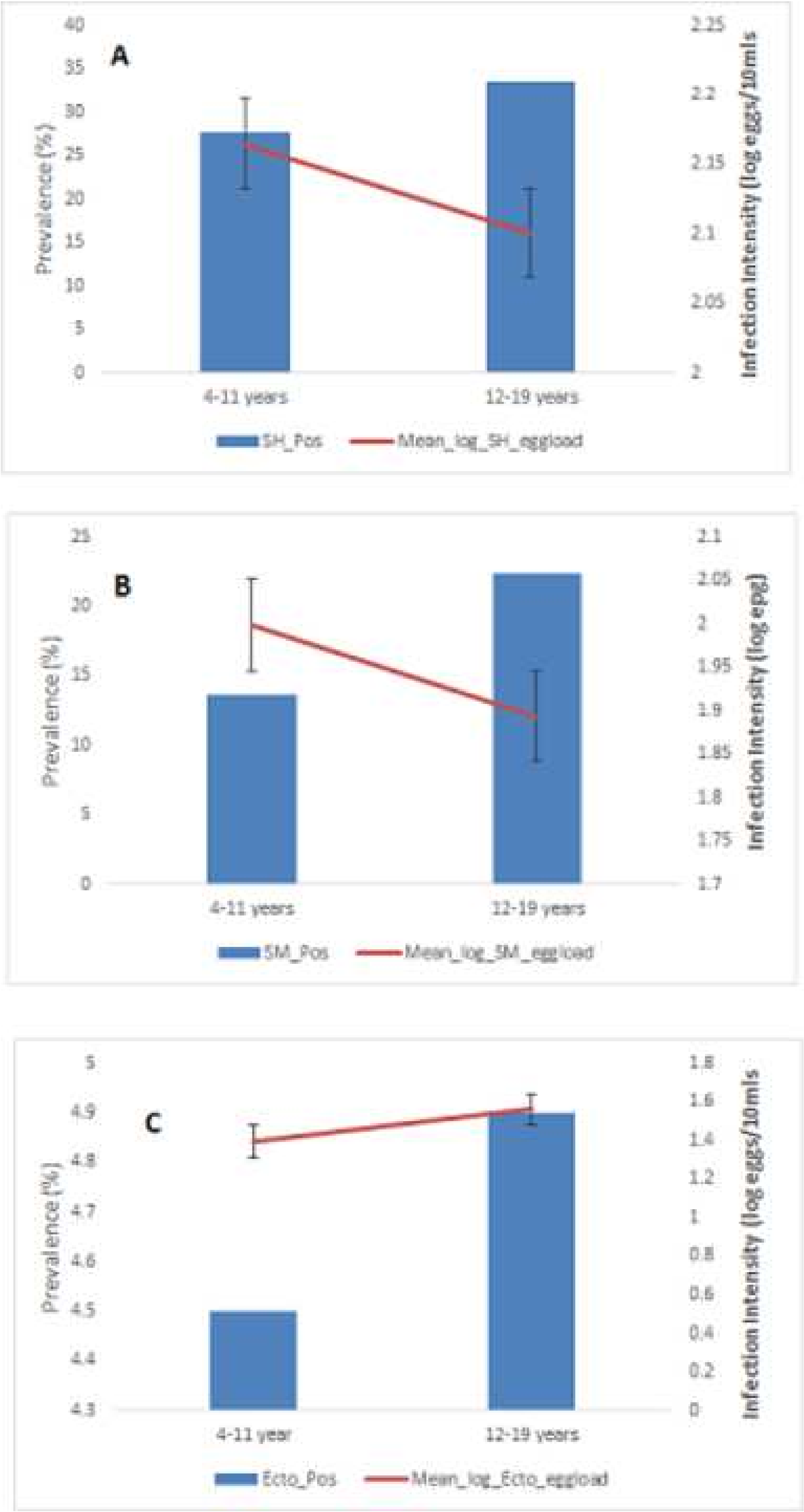

Figure 3

Click here to access/download;Figure;Ojo et al_Figure 3.tif

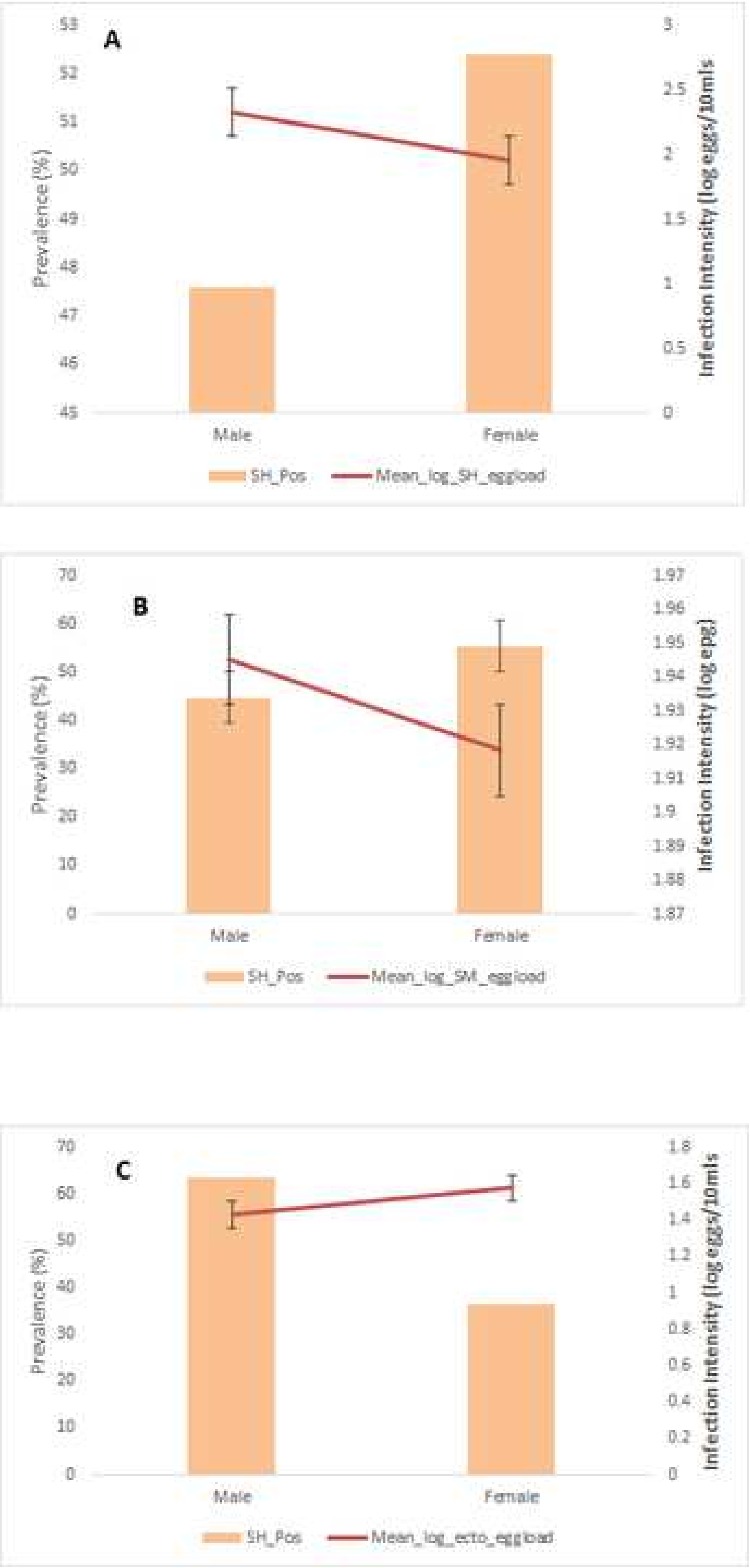

Table Click here to access/download;Table;Tables_Ojo et al_ectopic.docx

Table 1: General characteristics and prevalence of human schistosomiasis in the study population

Characteristics	Total Population	***Sh*** (single infection) n=89	***Sm*** (single infection) n=41	***Sh*/*Sm*** (Mixed infection) n=33	E***Sm*** (single infection) n=1	E***Sm*/*Sh*** (Mixed infection) n=10	***Sh*/*Sm*/** E***Sm*** (Mixed infection) n=11
**Mean age ± SD**	11.6±3.16	12.0±3.10	12.5±2.70	12.3±2.50	-	11.3±2.21	12.8±3.5
**Mean weight ± SD**	31.2±9.60	32.9±9.96	32.0±9.10	33.8±7.74		29.1±5.07	33.4±10.40
**Mean Height± SD**	1.41±0.78	1.41±0.16	1.69±1.98	1.40±0.15		1.37±0.07	1.39±0.16
**Age group**							
**4 – 11 years (%)**	221 (47.4)	40 (45.0)	15 (36.6)	11 (33.3)	0	6 (60.0)	4 (36.4)
**12 – 19 years (%)**	245 (52.6)	49 (55.1)	26 (63.4)	22 (66.7)	1 (100)	4 (40.0)	7 (63.6)
***p*-value**		*0.638*	*0.189*	*0.105*	-	*0.528*	*0.550*
**Sex**							
**Male (%)**	211 (45.3)	38 (42.7)	17 (41.5)	15 (45.5)	0	8 (80.0)	6 (54.5)
**Female (%)**	255 (54.7)	51 (57.3)	24 (58.5)	18 (54.5)	1(100)	2 (20.0)	5 (45.5)
***p*-value**		*0.636*	*0.627*	*1.000*	-	*0.050*	*0.556*
**Blood in Urine**							
**Present**	98 (21.0)	30 (33.7)	6 (14.6)	11 (33.3)	0	6 (60.0)	8 (72.7)
**Absent**	368 (79.0)	59 (66.3)	35 (85.4)	22 (66.7)		4 (40.0)	3 (27.3)
***p*-value**		*0.002**	*0.421*	*0.079*		*0.009**	*0.0003**

Key: *Sh*: *Schistosoma haematobium*; *Sm*: *Schistosoma mansoni*; E*Sm*: Ectopic *Schistosoma mansoni*

Table 2: Schistosomal infection prevalence and intensities

	***Sm* infection**		***Sm* infection**		**Prevalence n=466 (%)**	***Sh* infection intensity**		***Sm* infection intensity**	
	Urine	Stool	Urine	stool		GM egg/ 10 ml	(95% CI)	GM epg	(95% CI)
Positive participants					**185 (40.0)**				
**Single infection**	[illegible]	-	-	-	89 (19.1)	189.4	115.9-262.9		
	-	+	-	-	0				
	-	-	+	-	1 (0.2)				
	-	-	-	+	41 (9.0)			115.7	78.4-152.9
**Mixed Infections**					**44 (9.5)**	**668.6**	**395.4-941.8**	**229.2**	**100.5-357.9**
E***Sm*** Infection			+		**21 (4.7**)				
Negative participants					**281 (60.4)**				
Overall ***Sh*** infections					143 (30.8)	399.4	263.7-535.2		
Overall ***Sm*** infections					85 (18.3)			174.4	105.6-243.3

Key: *Sh*: *Schistosoma haematobium*; *Sm*: *Schistosoma mansoni*; E*Sm*: Ectopic *Schistosoma mansoni*

Table 3: Relation between ectopic Schistosoma mansoni eggs in urine and intensities of S. haematobium egg in urine and S.mansoni egg in stool.

**Intensities of *Sh* in urine (eggs/10ml)**	***Sm* eggs in urine**			
	N	Cases	Prevalence (%)	
0	323	1	0.3	
1-9	0	0	0	<0.0001
10-49	32	1	3.1	
≥ 50	111	20	18	
Total	466	22		
**Intensity of *Sm* in stool (epg)**				
0	381	11	2.9	
1-99	53	6	11.3	0.0003
≥ 100	32	5	15.6	
Total	466	22		
