# Supplementary material for: Prevalence of urogenital and intestinal schistosomiasis among school children in South-west Nigeria
Source: PLoS Negl Trop Dis. 2021 Jul 27;15(7):e0009628. doi: 10.1371/journal.pntd.0009628 (PMC8345861; doi:10.1371/journal.pntd.0009628)
Supplement: Attachment — Submitted filename: Response to reviwers comments.docx [file pntd.0009628.s002.docx]

PLOS Neglected Tropical Diseases

***********************

We appreciate the time and efforts of our reviewer. We are happy to submit our comments to their queries. All our comments are in red font. Thank you

Reviewer's Responses to Questions

**Key Review Criteria Required for Acceptance?**
As you describe the new analyses required for acceptance, please consider the following:

**Methods**
-Are the objectives of the study clearly articulated with a clear testable hypothesis stated?</br>
-Is the study design appropriate to address the stated objectives?</br>
-Is the population clearly described and appropriate for the hypothesis being tested?</br>
-Is the sample size sufficient to ensure adequate power to address the hypothesis being tested?</br>
-Were correct statistical analysis used to support conclusions?</br>
-Are there concerns about ethical or regulatory requirements being met?</br></br>

Reviewer #1: The methods used are ok, but my worry is on the sample collection.

Reviewer #2: The objectives of the study are not clear.

In the methods, the authors do not explain why the study was conducted only in school children. In the results, two groups are presented according to age, but in the methods this division is not presented.

The abstract and the introduction has been corrected to make the objective clearer. The objective is to “investigate the prevalence of mixed human schistosome infection, intensity, and possible occurrence of ectopic egg elimination”.

School children are most vulnerable to the infection and the risk is very high. The choice of school children has been included in the introduction Lin….

The collection of only one urine sample and one stool sample for each student may have limited the finding of positive samples in the study: why is this point not discussed by the authors?

We agree that this is a limitation, and it has been included in the study

The samples were collected from two communities, but the results are presented as a single sample. What is the reason for this choice? Was the prevalence similar in both communities? For both species of Schistosoma?

The two communities are within the same locations, only separated by the dam. The prevalence is similar in both. This is stated in the manuscript

In the statistical analysis, was a normality test applied to the sample to choose the appropriate comparison test between the variables.

Contingency table was used for the analysis in table 1. This does not require a normality test since it was based of frequencies of the presence or absence of the infection.

The comparison in table 3 has been removed because of the low counts (less than 5) of the number of people infected at different intensities.

It is known that the intensity of schistosoma infection is affected by different factors, and not just age and sex. Variables such as hygiene habits, number and time of exposure to water collections, sanitation, previous infection and previous parasitic treatment, could also have been analyzed in the epidemiological study. Why were they not analyzed?
The absence of morbidity analysis in co-infection is a limiting factor in the study.

We agree with this comments. These factors are well known as contributing factors to the epidemiology of schistosomiasis. The focus of our study is actually on the occurrence of both S. haematobium and S. mansoni and ectopic egg elimination. We hope in our future study to analyze the snail hosts and further investigate the presence of hybrid parasites in the host. We can also incorporate other variables as suggested.

The authors gave no information about the study period.

The study was conducted between 2019

Reviewer #3: The objectives of the study are not clearly articulated

The abstract and the introduction has been corrected to make the objective clearer. The objective is to “investigate the prevalence of mixed human schistosome infection, intensity, and possible occurrence of ectopic egg elimination”.

The study design is not described

The study is a cross-sectional study. It is now addressed in the study

Though the sample size is big enough, the population is only partially described
the sample size is big enough

The study population has now being well described in the methodology section

The statistical analysis is not appropriately reported

This has been corrected

There are no ethical issues

Thank you

--------------------
**Analysis**

-Does the analysis presented match the analysis plan?</br>
-Are the results clearly and completely presented?</br>
-Are the figures (Tables, Images) of sufficient quality for clarity?</br></br>

Reviewer #1: The analysis are okay and the results presented well. The issue is on the signs on table 2 which were not explained

Thank you the signs have been explained

Reviewer #2: The figures shown are not in high resolution. The variables that present significant results could be presented in the figure itself and not in the legend.

The figures has been formatted using PACE as recommended by the journal. For neatness and uniformity we opted to have the values as legend.

Table 3 is confused for the interpretation of the results.
It is necessary to explain the set of abbreviations: 'ectopic Sm/Sh' and 'ectopic Sm/Sh/Sm'
The key defining all the abbreviations has been inserted to all tables.

Reviewer #3: No specific comments

--------------------

**Conclusions**
-Are the conclusions supported by the data presented?
-Are the limitations of analysis clearly described?
-Do the authors discuss how these data can be helpful to advance our understanding of the topic under study?
-Is public health relevance addressed?

Reviewer #1: Authors should remove sections on hybridization as pointed out in the text

It is clearly stated that “Although our study did not conduct hybridization study, the elimination of ectopic *Sm* could imply hybridization between the two human schistosomes as previously reported in Cameroon [14, 15] that may warrant further investigation.” It is important that we retain this aspect of the discussion so that readers can appreciate the study and the need for further study. Thank you

All my other comments are inserted as notes in the text

Reviewer #2: When introducing the manuscript, the authors point out that hybridization between schistosoma species has not yet been reported in Africa (line 79). However, in lines 238 and 239 of the discussion, they present 2 studies that report the ectopic presence of schistosoma eggs in Cameroon (reference 14) and Senegal (reference 33). It may therefore be appropriate to rewrite the introduction paragraph.

Thanks for the observation. The sentence has been corrected

The discussion of the article should be reviewed. It is superficial and limited.
At the beginning of the article, the authors suggest that the encounter of ectopic eggs is unprecedented in the study area. But throughout the article, there is decreasing emphasis on this observation and the discussion does not present a differential result. If it is really an unprecedented result, I recommend modifying the article to highlight this finding.
We have improved the discussion based on this suggestion

As this is an epidemiological study, it would be interesting to add a paragraph on the importance of the findings for local public health, according to the guidelines for coping with schistosomiasis as an important public health problem by the WHO.

This has been addressed

Reviewer #3: The limitations are not clearly described

Thank you for the comment

It has been included in the text

--------------------

Editorial and Data Presentation Modifications?
Use  this section for editorial suggestions as well as relatively minor modifications of existing data that would enhance clarity. If the only modifications needed are minor and/or editorial, you may wish to recommend “Minor Revision” or “Accept”.

Reviewer #1: Minor revision

Reviewer #2: (No Response)

Reviewer #3: (No Response)

--------------------

Summary and General Comments
Use  this section to provide overall comments, discuss strengths/weaknesses of the study, novelty, significance, general execution and scholarship. You may also include additional comments for the author, including concerns about dual publication, research ethics, or publication ethics. If requesting major revision, please articulate the new experiments that are needed.

Reviewer #1: The study is good  and deserves publication, however the authors should revise the use of words "impact" since the study is basically about prevalence

Thank you this has been revised

Reviewer #2: (No Response)

Reviewer #3: The manuscript entitled "Epidemiology of mixed urogenital and intestinal schistosomiasis among school children in two endemic communities of Southern Nigeria"submitted by Ojo et al.  is important, and could give insight into the Schistosomiasis control challenges in areas where both major species are endemic.

However, the manuscript has a substantial limitations which the authors have to address prior any further consideration.

While the title clearly indicates an epidemiological study, there is no clear study design and nor any sampling frame which may permit a validation of the findings. The sites description indicated a previous study reporting the presence of S. hematobium, in urine and not the S. mansoni species. The statistical test used to test association, is not reported appropriately. All together the manuscript requires a deep revision.

In details:

Abstract section:
It is not clear what the authors called "ectopic egg": for non-experts in schistosomiasis a brief explanation is required here.

This has been included

The study design and methodology are missing, as well as brief study procedures, as appropriate in the Abstract.

The study design is cross-sectional and this has been included. Brief procedure is also included.

Prevalence and intensity are confusedly reported and should be separated.

This has been done

Would hematuria have been associated with *S. hematobium*?

Yes. This is also what we observed in the study

Introduction Section:

•       There is poor background information regarding clinical or epidemiological consequences of co-infection of S.h /S.m as well as ectopic eggs. Please provide more information to justify the research question.

We have provided more background information on co-infection. In addition more information on the consequences of co-infection was discussed in the discussion

MM section:
•       Study design is missing

Included in text
•       Sample size calculation and sample collection should be separated

It has been addressed
•       Study procedure as well as inclusion and exclusion criteria are missing

It has been included in the text
•       While the sample size calculated was 289, the authors recruited 466 participants: is there an explanation?

The sample size of 289 was the minimum sample unit require at a given level of power (80%). Higher sample size above the calculated values is better

•       Is there any reason for use of sterile container to collect stool and urine?

No special reason is necessary to prevent contamination

Results section:
•       What does '5gm' mean?\

It was an error it has therefore been removed
•       Will mixed infection Sh/Sm differ from Sm/Sh? No
•       Can author provide the odds CI: 95% of hematuria in S.h positive versus S.h negative?. (CI 95% 1.39-3.86; OR = 2.3)

Discussion Section
1.      Authors should cross check the p-value which should be <0.05 instead of >0.05

It has been corrected
2.      Authors should provide more specific information on lower liver morbidity and higher bladder morbidity

Lowering of liver morbidity occurs in mixed infection as result of ectopic egg elimination. For example the deposition of *S. mansoni* egg in the urinary oviposition site will reduce the number of classical S. mansoni egg that can cause liver morbidity. This has been discussed with appropriate references

3.      What do the authors mean by this: "Consequently, the collection of urine specimens and their examination may not be necessary in the classification of communities according to the level of endemicity of urinary schistosomiasis".?

This statement has been revised. We are looking at a situation where haematuria could be used to determine the endemicity of urinary schistosomiasis in the absence of urine microscopy.
--------------------
PLOS authors have the option to publish the peer review history of their article (<a href="<https://journals.plos.org/plosntds/s/editorial-and-peer-review-process#loc-peer-review-history>" target="_blank">what does this mean?</a>). If published, this will include your full peer review and any attached files.<br><br>

If you choose “no”, your identity will remain anonymous but your review may still be made public.<br><br>

<b>Do you want your identity to be public for this peer review?</b> For information about this choice, including consent withdrawal, please see our <a href="<https://www.plos.org/privacy-policy>" target="_blank">Privacy Policy</a>.

Reviewer #1: Yes: Prof Sammy Olufemi Sam-Wobo

Reviewer #2: No

Reviewer #3: No


Figure Files:
While revising your submission, please upload your figure files to the Preflight Analysis and Conversion Engine (PACE) digital diagnostic tool, [https://pacev2.apexcovantage.com](https://pacev2.apexcovantage.com/). PACE helps ensure that figures meet PLOS requirements. To use PACE, you must first register as a user. Then, login and navigate to the UPLOAD tab, where you will find detailed instructions on how to use the tool. If you encounter any issues or have any questions when using PACE, please email us at [figures@plos.org](mailto:figures@plos.org).
 
Data Requirements:
Please note that, as a condition of publication, PLOS' data policy requires that you make available all data used to draw the conclusions outlined in your manuscript. Data must be deposited in an appropriate repository, included within the body of the manuscript, or uploaded as supporting information. This includes all numerical values that were used to generate graphs, histograms etc.. For an example see here: <http://www.plosbiology.org/article/info%3Adoi%2F10.1371%2Fjournal.pbio.1001908#s5>.
 
Reproducibility:
To enhance the reproducibility of your results, PLOS recommends that you deposit laboratory protocols in [protocols.io](http://protocols.io/), where a protocol can be assigned its own identifier (DOI) such that it can be cited independently in the future. For instructions see <https://journals.plos.org/plosntds/s/submission-guidelines#loc-methods>
