# Supplementary material for: Prevalence of urogenital and intestinal schistosomiasis among school children in South-west Nigeria
Source: PLoS Negl Trop Dis. 2021 Jul 27;15(7):e0009628. doi: 10.1371/journal.pntd.0009628 (PMC8345861; doi:10.1371/journal.pntd.0009628)
Supplement: Attachment — Submitted filename: Response to reviwers comments_Minor V2.docx [file pntd.0009628.s004.docx]

Dear Editor,

The few minor alterations have been attended to. The highlighted version of the manuscript contain the few responses. Many thanks

- All authors name are spelled correctly in the manuscript file. I do not have access to the submission form. The correct arrangement for first author is Johnson A. Ojo
- An author summary has been provided

Olusola Ojurongbe
